# Correlation analysis of NT-proBNP (N-terminal probrain natriuretic peptide), 25-Hydroxyvitamin D, HMGB1(High-mobility group box 1), ACTA (endogenous activin A), blood glucose level, and electrolyte level with developmental quotient scores in neonates with hypoxic-ischemic encephalopathy

**DOI:** 10.1186/s12887-022-03606-6

**Published:** 2022-12-28

**Authors:** Guiling Liu, Sisi Cheng, Li Wan, Yanyan Li, Qian Zhao, Jianxin Liu, Xiufang Jiang

**Affiliations:** 1 Department of Pediatrics, The First Hospital of Hebei Medical University, No. 89 Donggang Road, Yuhua District, Shijiazhuang, 050031 China; 2 Shijiazhuang Center for Disease Control and Prevention institute of Epidemic Diseases, Shijiazhuang, China

**Keywords:** N-terminal probrain natriuretic peptide, 25-hydroxyvitamin D, High-mobility group box 1, Endogenous activin A, Blood glucose level, Hypoxic-ischemic encephalopathy, Developmental quotient scores, Neonates

## Abstract

**Background:**

To investigate the correlation between N-terminal probrain natriuretic peptide (NT-proBNP), 25-hydroxyvitamin D (25-(OH) D), high-mobility group box 1(HMGB1), endogenous activin A (ACTA), blood glucose level, electrolyte levels and developmental quotient (DQ) scores of Hypoxic-ischemic encephalopathy (HIE).

**Methods:**

In this retrospective study, a total of 90 neonates diagnosed with HIE who were admitted to our hospital from January 2018 to June 2021 were retrospectively enrolled, and 40 healthy full-term neonates born in our hospital during the same period were randomly selected. Neonates with HIE and healthy conditions were set as the study group and control group, respectively. Neonates with HIE are divided into three subgroups, mild, moderate, and severe, based on the severity of HIE. The Gesell Developmental Scale (GDS) was used to assess neural development of neonates at 9 to 12 months postnatal. Biomarkers of peripheral venous blood were measured and collected in all neonates, including NT-proBNP, (25-(OH) D), HMGB1, ACTA, electrolyte levels and blood glucose levels. General demographic information and Apgar score were compared between the two groups. The differences between the two groups of biomarkers were compared and the correlation between these biomarkers and DQ scores was evaluated.

**Results:**

There was no significant difference in gestational age, maternal age, gender, way of birth, birth weight, gestational age and whether the mother was a primipara between the two groups (*P*>0.05). The 10 min Apgar score of the study group (5.87±0.36) was lower than that of the control group (9.37±0.32) with significant difference (*P*<0.05). The levels of NT-proBNP, HMGB1, and ACTA in the study group were higher than that in the control group (243.87±21.29 pmol/L vs. 116.98±22.19 pmol/L; 8.92±1.87 μg/L vs. 3.28±1.08 μg/L; 23.78±0.89 ng/ml vs. 2.98±0.38 ng/ml), while the levels of 25-(OH) D and electrolyte levels were lower than that in the control group (24.28±1.87 vs. 31.29±1.93; K+: 4.49±0.23 mmol/L vs. 4.73±0.21 mmol/L; Na+: 118.76±13.02 mmol/L vs. 134.28±12.29 mmol/L; Ca2+: 1.77±0.23 mmol/L vs. 2.35±0.26 mmol/L; Mg2+: 0.61±0.17 mmol/L vs. 0.91±0.17 mmol/L), with statistically significant differences (*P*<0.001). The levels of NT-probNP, HMGB1, ACTA and the incidence of hypoglycemia were the highest in the severe group, which were significantly higher than those in the moderate group and mild group (P<0.05). The levels of NT-probNP, HMGB1, ACTA and the incidence of hypoglycemia were the lowest in the mild group. The 25-(OH) D level, the incidence of hyperglycemia and electrolyte levels were the lowest in the severe group, which were significantly lower than those in the moderate and mild groups (all *P*<0.05). Meanwhile, the 25-(OH) D level, the incidence of hyperglycemia and electrolyte levels in the moderate group were lower than those in the mild group, and the differences were statistically significant (all *P*<0.05).

The incidence of hyperglycemia in severe group (16 cases) was the lowest, significantly lower than that in moderate group (17 cases) and mild group (22 cases), and the difference was statistically significant (all *P*<0.05).

The DQ scores of HIE neonates were negatively correlated with NT-proBNP, HMGB1, and ACTA (*r*=-0.671, -0.421, -0.518, all *P*< 0.001). The DQ scores was positively correlated with levels of 25-(OH) D and blood glucose level (*r* =0.621, 0.802, all *P*< 0.001). The DQ scores was also positively correlated with levels of potassium, sodium, calcium and magnesium (0.367, 0.782, 0.218, 0.678, all *P*<0.001).

**Conclusion:**

The NT-proBNP, HMGB1, ACTA, 25-(OH) D, blood glucose levels and electrolyte levels are correlated with the severity of HIE, and developmental quotient scores in neonates with HIE. These biomarkers are suggestive for assessing the prognosis of neonate with HIE.

## Background

Hypoxic-ischemic encephalopathy (HIE) refers to hypoxic-ischemic damage to the brain caused by perinatal asphyxia, which can lead to abnormal neurological development and is an important cause of permanent neurological deficits and even death in neonates [1]. HIE has a high incidence, about 30% of children are left with lifelong neurological damage such as cerebral palsy, epilepsy, and visual impairment, which seriously affects the quality of life of neonates [2]. Most of treatments for HIE belong to comprehensive symptomatic treatment and the therapeutic effect is not satisfactory enough [3]. Hence, exploring the prognostic factors of neonates with HIE plays a positive role in finding therapeutic targets and improving therapeutic effects.

The current diagnostic methods of HIE mainly include magnetic resonance imaging (MRI), computed tomography (CT), ultrasound, electroencephalographic (EEG), and Sarnat score [4]. Sarnat scores are subjective, and the use of imaging tests requires about 72 hours after the onset of neonate hypoxic-ischemic encephalopathy, which is not conducive to early monitoring [5]. Early detection and timely treatment are essential to improve prognosis [6]. Therefore, it has been a research focus to explore biomarkers that are more convenient and have early warning value [7]. Studies have shown that cytokine-induced cascade reactions can occur in the pathological process of HIE [8]. Understanding the role of cytokines in the pathological development of HIE is of great significance for the early diagnosis and prognosis evaluation of the disease.

High-mobility group box 1 (HMGB1) is a mediator of early response, and as a damage-related protein mediates neuroinflammation and brain injury in a variety of neurological diseases. Previous studies suggested that HMGB1 was an activation signal of nervous system inflammation [9]. There is a close risk relationship between N-terminal probrain natriuretic peptide (NT-proBNP) and cerebrovascular diseases. It was reported that NT-proBNP had important significance in the diagnosis and prognosis of acute and chronic heart failure [10]. Evaluation of the level of NT-proBNP in the occurrence of HIE has potential application value for early neurological intervention while paying attention to the protection of cardiac function and maintaining the stability of cardio-cerebral blood perfusion. 25-HydroxyVitamin D [(25-(OH) D)] is an important part of normal neuronal development and plays an important role in neonates' responses to brain injury [11]. Endogenous Activin A (ACTA) is an important group of cytokines discovered in recent years. Studies suggested that ACTA had anti-inflammatory and neuroprotective effects, and was beneficial to repairing interstitial cells [12, 13]. The blood sugar provided by the placenta stops immediately after the birth of the newborn, and the stability of blood sugar in the body mainly depends on the stored liver glycogen. HIE can cause metabolic disorders, so abnormal blood glucose often occurs in neonates with HIE. Since blood glucose is an important energy source for nervous tissue, metabolic disorders caused by HIE will also affect neurological function to different degrees [14]. In addition, recent studies have pointed out that electrolyte disorder may be closely related to the occurrence and prognosis of a variety of cardiovascular and cerebrovascular diseases [14]. All the above studies indicate that biomarkers play an important role in the occurrence and development of HIE. However, there are few clinical studies on the correlation between NT-proBNP, (25-(OH) D, HMGB1, ACTA, blood glucose level, electrolyte levels and DQ scores of neonates with HIE. Hence, the aim of this study was to explore the correlation between NT-proBNP, 25-(OH)D, HMGB1, ACTA, blood glucose level and neurodevelopmental prognosis in neonates with HIE.

## Materials and methods

### Study patients

In this retrospective study, a total of 90 neonates with HIE admitted to our hospital from January 2018 to June 2021 and 40 healthy full-term neonates born in our hospital during the same period were enrolled. HIE neonates were set as the study group and healthy neonates as the control group.

Inclusion criteria: (1) HIE neonates met the relevant diagnostic criteria in neonate hypoxic-ischemic encephalopathy and were were diagnosed within 6 hours of birth [15]; (2) HIE neonates all had neurological symptoms at birth and were hospitalized within 24 hours after onset; (3) HIE neonates who underwent brain CT examination before receiving mild hypothermia and the results showed low-density focal changes which were suggestive of brain necrosis; (4) Informed consent signed by family members and guardians.

Exclusion criteria: (1) Convulsive neonates due to intracranial hemorrhage, birth injury and electrolyte disturbance; (2) Complications of nervous system infection; (3) Puerpera with abnormal placental function; (4) Neonates with congenital diseases; (5) Neonates with brain damage caused by intrauterine infection; (6) Neonates with other types of brain disease.

Informed consent was obtained from all subjects and/or their legal guardian(s). This study was approved by the institutional review boards of the First Hospital of Hebei Medical University. All data were handled according to the ethical standards of the Declaration of Helsinki

### Evaluation of HIE severity

The severity of HIE was classified according to the following criteria [16]: (1) Mild, neonate muscle tone continued to decrease or nerve excitability increased within 72 hours after birth, but convulsion did not develop; (2) Moderate, lethargy within 72 hours after birth accompanied by hypotonia, weakened primitive reflex or convulsion; (3) Severe, frequent convulsions, apnea, inactivity or coma within 72 hours after birth.

### General Information Collection

The gender, age of the mother, whether the mother was a primipara or not, gestational age, birth weight, and 10 min Apgar score of the neonates were collected. Apgar score included five objective signs, including heart rate, skin color, response to stimulus, respiration, and muscle tension. The score of each sign was 0 ~ 2 points, and each score was added to the total Apgar score.

### Biomarker detection

Venous blood of 5ml was collected 24 hours after birth from neonates in the study group and the control group. The serum was centrifuged (2500r/min, 15min) with a centrifugal radius of 4.5cm. Enzyme-linked immunosorbent assay was used to determine NT-proBNP (The kits were produced by Wuhan Easy Diagnosis Biomedicine Co., Ltd, China), HMGB1(The kits were produced by Cell Signaling, USA), and ACTA (The kits were produced by Shanghai i-Reader biological technology Co., Ltd, China). Potassium, sodium, calcium, and magnesium are determined by the enzymatic method. Potassium, sodium, calcium, and magnesium are determined by the enzymatic method. The automatic biochemical analyzer was produced by Roche (Cobas8000, USA). The 25- (OH) D was determined by electrochemiluminescence (The kits were produced by Dyets, USA). The blood glucose level was measured with the blood glucose meter and matching test paper produced by Johnson & Johnson company of the United States; Glycosylated hemoglobin (HbA1c) was measured by Mindray H50 glycosylated hemoglobin analyzer. Assessment criteria for blood glucose levels [17]: (1) Peripheral blood glucose > 150mg/dL was considered hyperglycemia; (2) Peripheral blood glucose ≤40 mg/dL was considered hypoglycemia.

### Assessment of neurodevelopmental status

The prognosis of neurodevelopment was assessed by Gesell Development Scale (GDS) [18] and the assessment was performed by two experienced pediatric rehabilitation physicians with more than 5 years of experience at 9 to 12 months postnatal. The two physicians were blinded to general information of neonates at the time of evaluation. If there was disagreement between two physicians, a third chief physician with more than 20 years' experience would make the final judgment and the evaluation of the neurodevelopmental status of the neonates would take the opinions of the third chief physician as reference. The developmental quotient (DQ) scores on five Gesell subscales (gross motor, fine motor, adaptive behavior, language, and social behavior) were recorded. DQ = developmental age/actual age.

The criteria of intelligence development were :(1) developmental retardation was defined when the developmental quotient of one or more energy regions < 75 points; (2)75-85 scores were considered a marginal state, and (3) > 85 scores were considered as well developed. The sum of the scores of the five subscales was the total developmental quotient, and the higher the total developmental quotient, the better the prognosis of neurodevelopmental children.

### Statistical analysis

Statistical analysis was performed using the SPSS software program (version 21.0; IBM Corp, Chicago, IL, USA). Normally distributed measurement data were expressed as mean ± standard deviation (SD). The Student's t-test was used for comparison of measurement data between two groups, and one-way ANOVA was used for comparison between multiple groups. A Chi-square test was used to compare the gender between the two groups. Pearson correlation was used to evaluate the correlation between the DQ scores and biomarkers in HIE. The test level α was 0.05 on both sides. *P* < 0.05 was considered statistically significant.

## Results

### General data

The study group included 49 males and 41 females with a mean gestational age of 38.87±1.23 weeks (ranging from 37 to 41 weeks). The control group included 21 males and 19 females, with a mean gestational age of 39.21±1.35 weeks (ranging from 37 to 42 weeks). There was no significant difference in gestational age, maternal age, gender, way of birth, birth weight, gestational age and whether the mother was a primipara between the two groups (*P*>0.05). The Apgar score of the study group (5.87±0.36) was lower than that of the control group (9.37±0.32), and the difference was statistically significant (*P* < 0.05). In the study group, the number of patients with mild, moderate and severe HIE was 40, 31 and 19, respectively. (Table 1).

**Table 1:** Comparison of general demographic data between the two groups

Item	Study group (*n*=90)	Control group (*n*=40)	t/χ^2^	*P*
Gestational age (weeks)	38.87±1.23	39.21±1.35	-1.411	0.161
Mother's age (year)	28.98±5.29	29.21±5.63	-0.205	0.838
Gender (male/female)	49/41	21/19	1.991	0.158
Way of birth (n)			0.033	0.855
Eutocia	57	26		
Cesarean	33	14		
Birth weight (g)	3456.19±451.21	3582.31±490.25	-1.432	0.155
Primipara cases (n)	73	32	0.022	0.882
Severity of HIE				
Mild	40			
Moderate	31			
Severe	19			
Apgar score in 10 minutes	5.87±0.36	9.37±0.32	-36.611	<0.001
Cardiopulmonary resuscitation (n,%)	32(35.56%)	-		

*HIE* hypoxic-ischemic encephalopathy

### Comparison of biomarker levels

The levels of NT-proBNP, HMGB1, ACTA, incidence of hypoglycemia and hyperglycemia in the study group were higher than that in the control group, while levels of 25-(OH) D and and electrolyte levels were lower than that in the control group, the differences were statistically significant (all *P* < 0.001) (Table 2).

**Table 2:** Comparison of NT-probNP, 25-(OH)D, HMGB1, ACTA, blood glucose level and electrolyte level between the two groups (mean±SD)

Item	Study group (*n*=90)	Control group (*n*=40)	t	*P*
NT-ProBNP (pmol/L)	243.87±21.29	116.98±22.19	28.704	<0.001
25-(OH) D (ng/ml)	24.28±1.87	31.29±1.93	-19.373	<0.001
HMGB1(μg/L)	8.92±1.87	3.28±1.08	18.325	<0.001
ACTA (ng/ml)	23.78±0.89	2.98±0.38	151.275	<0.001
Blood glucose (n,%)			38.519	<0.001
Incidence of hypoglycemia	52(57.78)	-		
Incidence of hyperglycemia	38(42.22)	-		
Electrolyte				
K^+^(mmol/L)	4.49±0.23	4.73±0.21	-5.372	<0.001
Na^+^(mmol/L)	118.76±13.02	134.28±12.29	-6.039	<0.001
Ca^2+^(mmol/L)	1.77±0.23	2.35±0.26	-11.608	<0.001
Mg^2+^(mmol/L)	0.61±0.17	0.91±0.17	-8.686	<0.001

*NT-ProBNP* N-terminal pro-brain natriuretic peptide, *25-(OH) D* 25-Hydroxyvitamin D, *HMGB1* High-mobility group box 1, *ACTA* endogenous activing A, *DQ* developmental quotient

### The relationship between biomarkers and HIE severity

The levels of NT-proBNP, HMGB1 and ACTA in the severe and moderate groups were higher than those in the mild group, while 25-(OH) D and electrolyte levels were lower than those in the mild group, the differences were statistically significant (*P* <0.001). The levels of NT-proBNP, HMGB1 and ACTA in the severe group were higher than those in the moderate group, while the 25-(OH) D and electrolyte levels were lower than those in the moderate group, with statistically significant differences (*P* <0.001), The incidence of hyperglycemia in severe group (16 cases) was the lowest, significantly lower than that in moderate group (17 cases) and mild group (22 cases), and the difference was statistically significant (all *P*<0.05), as shown in Table 3.

**Table 3:** Comparison of NT-probNP, 25-(OH) D, HMGB1, ACTA, blood glucose level and electrolyte level in neonates with different severity of hypoxic-ischemic encephalopathy (mean±SD)

Item	Mild(*n*=40)	Moderate(*n*=31)	Severe(*n*=19)	F	*P*
NT-ProBNP (pmol/L)	223.28±25.29	241.87±22.76^#^	268.92±23.28^#^*	10.405	<0.001
25-(OH) D (ng/ml)	26.87±2.22	22.09±2.19^#^	16.37±2.07^#*^	153.657	<0.001
HMGB1(μg/L)	6.27±1.68	8.97±1.72^#^	10.93±1.67^#*^	19.539	<0.001
ACTA (ng/ml)	17.82±0.93	23.27±0.91^#^	27.98±0.97^#*^	308.43	<0.001
Blood glucose (n,%)				10.067	0.007
Hypoglycemia	22	17	16^#*^		
Hyperglycemia	29	14	3^#*^		
Electrolyte					
K^+^(mmol/L)	4.56±0.14	4.41±0.23^#^	4.27±0.18^#*^	5.218	<0.001
Na^+^(mmol/L)	125.33±12.09	119.83±12.34^#^	103.29±12.18^#*^	10.264	<0.001
Ca^2+^(mmol/L)	2.07±0.21	1.73±0.19^#^	1.48±0.17^#*^	25.036	<0.001
Mg^2+^(mmol/L)	0.76±0.10	0.63±0.09^#^	0.51±0.12^#*^	16.134	<0.001

*NT-ProBNP* N-terminal pro-brain natriuretic peptide, *25-(OH) D,* 25-Hydroxyvitamin D, *HMGB1* High-mobility group box 1, *ACTA* endogenous activin A. ^#^, Compared with mild group with significant difference (*P*<0.05); ^*^, Compared with moderate group with significant difference (*P*<0.05)

### Correlation analysis of neurodevelopmental prognosis and biomarker in HIE

**Fig. 1:**
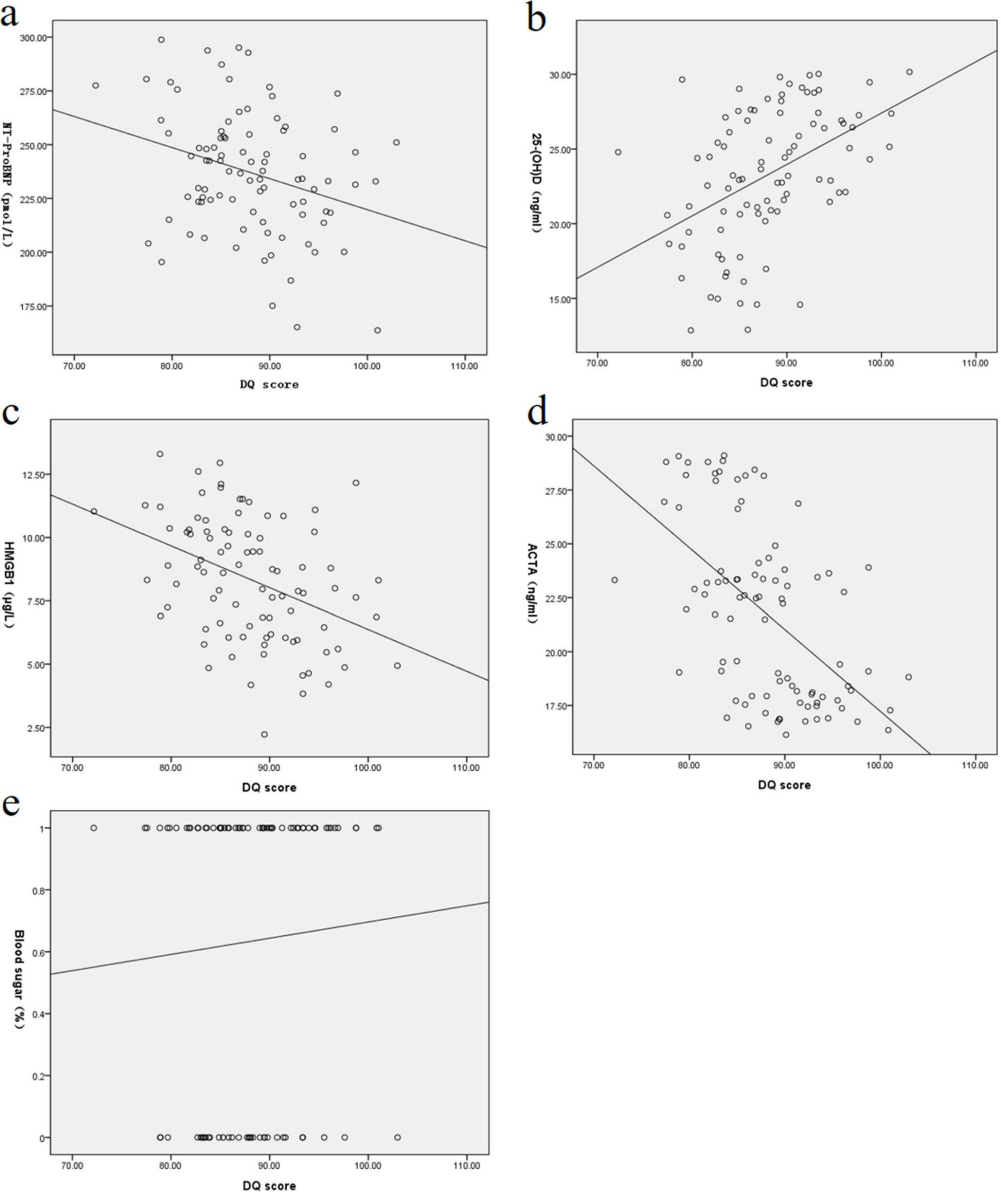
Scatter plot of correlation between biochemical indexes and DQ values **a.** Nt-probnp was negatively correlated with DQ score (*r*=-0.671, *P* < 0.001); **b.** 25-(OH) D was positively correlated with DQ score (*r*=0.621, *P* < 0.001); **c.** HMGB1 was negatively correlated with DQ score (*r*=-0.421, *P* < 0.001); **d.** ACTA was negatively correlated with DQ score (*r*= -0.518, *P* < 0.001); **e.** blood glucose level was positively correlated with DQ score (*r*=0.802, *P* < 0.001)

**Fig. 2:**
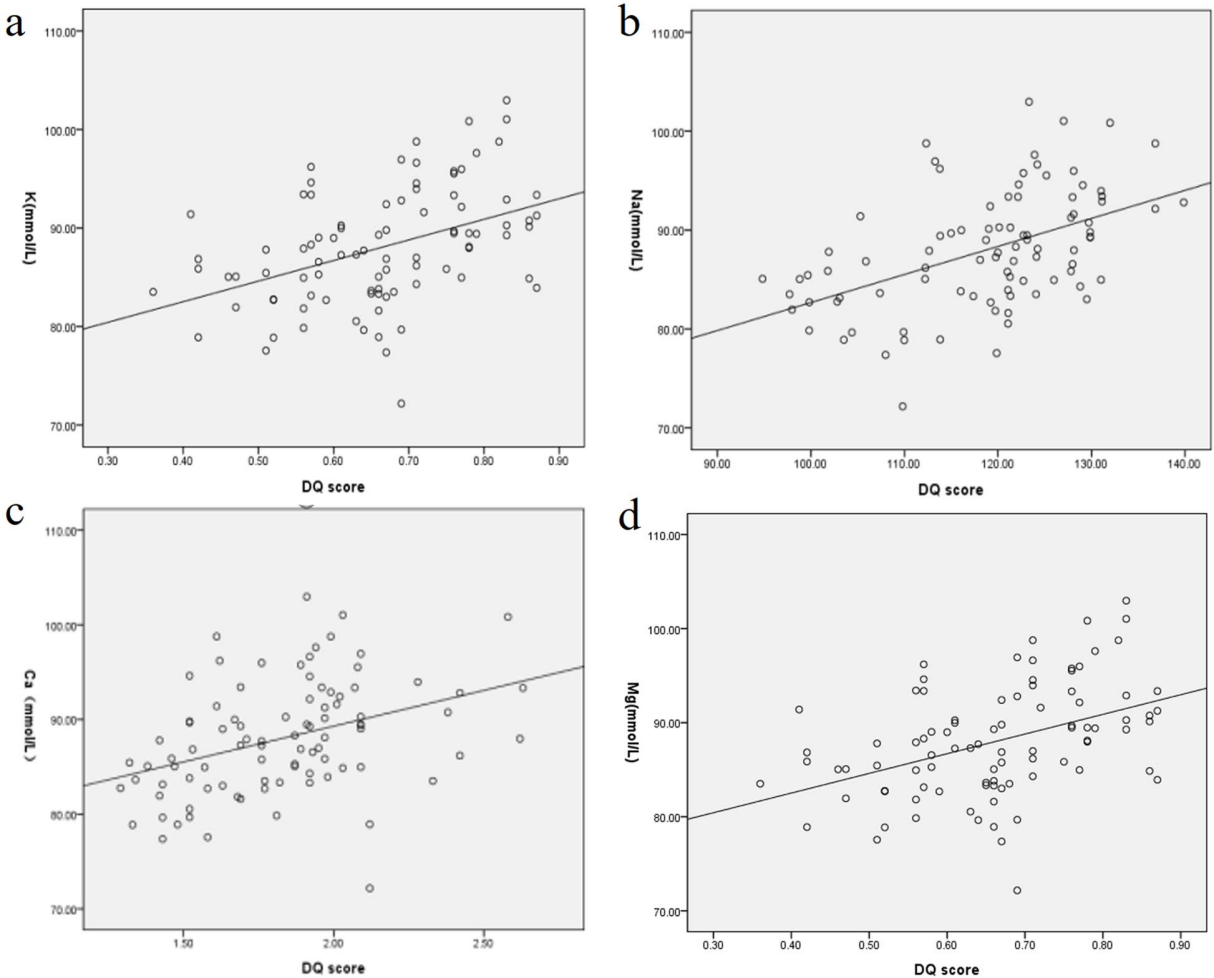
Scatter plot of correlation between electrolyte levels and DQ values. **a.** the potassium was positively correlated with DQ score (r=0.367, *P* < 0.001); **b.** the sodium was positively correlated with DQ score (r=0.782, *P* < 0.001); **c.** the calcium was positively correlated with DQ score (r=0.218, *P* < 0.001); **d.** the magnesium was positively correlated with DQ score (r= 0.678, *P* < 0.001)

In the study group, the DQ scores of mild, moderate and severe neonates were 92.19±5.29, 87.76±5.32 and 83.38±5.02, respectively. The DQ score of neonates with HIE was positively correlated with the levels of 25-(OH) D and blood glucose level (*r*=0.621, 0.802, respectively, *P* < 0.001). Pearson correlation analysis showed that the DQ scores of neonates with HIE were negatively correlated with NT-proBNP, HMGB1, and ACTA (*r*=-0.671, -0.421, -0.518, *P* < 0.001) (Fig. 1). The DQ scores was positively correlated with levels of potassium, sodium, calcium and magnesium (0.367, 0.782, 0.218, 0.678, all *P*<0.001) (Fig. 2).

## Discussion

Neonatal HIE is a central nervous system disease caused by fetal distress in utero or perinatal asphyxia. If the child does not receive timely and effective treatment, it will cause brain damage, even neurological sequelae, or lead to death [19]. HIE has an optimal time window for treatment before it progresses to permanent brain injury. The secondary damage to the nervous system can be avoided if the nerve is effectively protected within this time period [3]. Therefore, it is extremely important to actively explore serum markers related to HIE and apply them in the assessment of HIE patients for timely treatment intervention.

The pathological process of HIE involves the participation of a variety of biomarkers. Recent studies point out that there is a certain correlation between NT-proBNP and cerebrovascular diseases, and the higher the level of serum NT-proBNP in patients with hemorrhagic stroke, the more serious the neurological damage patients [20]. It was also pointed out that HIE combined with myocardial injury could lead to the elevation of plasma NT-proBNP level in the acute phase, which was helpful for the early diagnosis of myocardial injury and the judgment of neonate HIE [20]. A previous study pointed out that NT-proBNP, as a marker for the diagnosis of heart failure, could be used to diagnose heart failure diagnosis, treatment, and prognosis evaluation [21]. Other studies reported that serum NT-proBNP had a certain predictive value for the degree of neurological impairment and prognosis in patients with acute stroke [22]. 25-(OH) D is the main form of vitamin D in the body, and low levels of 25-(OH)D can cause brain damage and cognitive impairment. Lowe et al. [23] pointed out that the maternal vitamin D level of infants with HIE was low, suggesting that vitamin D was closely related to the occurrence and development of HIE. HMGB1 is a highly conserved non-histone protein in eukaryotic nuclei, widely expressed in glial cells and endothelial cells, and has biological functions such as regulating gene transcription, stabilizing cell nuclear structure, and mediating inflammatory response [24]. It was reported that HMGB1 plays an important role in the inflammatory response. When the craniocerebral injury occurs, HMGB1 is transferred from the nucleus to the cytoplasm and then to the extracellular region and promotes the destruction of the blood-brain barrier, inducing the inflammatory response after craniocerebral injury [24]. Lin et al. [25] pointed out that increased serum HMGB1 level was related to the severity of HIE. ACTA belongs to the hyper-transforming growth factor family, which has a variety of cellular functions, especially anti-inflammatory activity, and plays an important role in tissue damage and inflammatory repair [26]. The neonate behavioral neurological assessment (NBNA) scores were negatively correlated with HIE, and dynamic monitoring of serum ACTA levels was conducive to the prediction of sequelae [26]. The results of the present study showed that the levels of NT-proBNP, HMGB1, and ACTA in the study group were higher than that in the control group, while the levels of 25-(OH) D was lower than that in the control group (*P* < 0.001), suggesting that biological factors have a prompt role in the disease of HIE.

Previous studies have pointed out that neonates with HIE may suffer from glucose metabolism disorders due to energy metabolism disorders. Neonates with HIE are prone to stress hypoglycemia or hyperglycemia, and the blood glucose level of children is related to the prognosis [27]. Other study pointed out that clinicians should closely monitor the blood glucose level of neonates with hypoxic-ischemic encephalopathy and provide timely treatment measures for neonates with HIE, so as to improve their prognosis [28]. The results of this study suggest that the incidence of hypoglycemia and hyperglycemia in the experimental group was higher than that in the control group. This result is consistent with previous studies, which proves that children with HIE are prone to abnormal blood glucose and electrolyte levels, and blood glucose and electrolyte levels should be used as a common indicator to evaluate the condition of newborns in clinical practice.

The GDS system is a comprehensive developmental scale for infants and young children, used to evaluate the integrity of children's neuromotor, functional maturity, and potential for intellectual development, can objectively reflect the law of children's neuromotor and psycho-psychological development, and can be used as a diagnostic tool for neuromotor injury and intellectual impairment [29]. In this study, the GDS was used to evaluate the neurodevelopmental status of neonates with HIE. The results of this study showed that the DQ scores of neonates with HIE was negatively correlated with levels of NT-proBNP, HMGB1, and ACTA, but was positively correlated with levels of 25-(OH) D. The results of this study suggested that the neurodevelopmental status of children could be indirectly suggested by biological factors, which is suggestive for early intervention by clinicians.

This study also had the following limitations: The first is the limited sample size of the study. The levels of the biological factors involved in this study were not dynamically observed. Secondly, the cases included in this study were from a single center, so there may be some bias in the selection of patients. Thirdly, the follow-up period is relatively short. Therefore, a more scientific sample size and more comprehensive design are still needed to improve the quality of research results.

## Conclusion

The levels of NT-proBNP, HMGB1, ACTA, 25-(OH) D, blood glucose level and electrolyte levels were correlated with the severity and neurodevelopment of HIE. These biochemical indicators are helpful to assess the prognosis of HIE.

## Data Availability

All data generated or analysed during this study are included in this published article.

## Abbreviation

25-(OH) D: 25-hydroxyvitamin D
ACTA: endogenous activin A
CT: computed tomography
DQ: developmental quotient
EEG: electroencephalographic
GDS: Gesell Developmental Scale
HIE: Hypoxic-ischemic encephalopathy
HMGB1: high-mobility group box 1
MRI: magnetic resonance imaging
NT-proBNP: N-terminal probrain natriuretic peptide
